# Statistical optimization of arachidonic acid synthesis by *Mortierella alpina* CBS 754.68 in a solid‐state fermenter

**DOI:** 10.1002/fsn3.2667

**Published:** 2021-11-30

**Authors:** Zahra Ghobadi, Zohreh Hamidi‐Esfahani, Mohammad Hossein Azizi

**Affiliations:** ^1^ 41616 Department of Food Science and Technology Faculty of Agriculture Tarbiat Modares University Tehran Iran

**Keywords:** arachidonic acid, *Mortierella alpina*, Plackett‐Burman design, response surface methodology, solid‐state fermenter, sunflower oil cake

## Abstract

Arachidonic acid (ARA) is an omega‐6 fatty acid that plays a major role in human health. The present study optimizes the production of ARA by the soil fungus *Mortierella alpina* CBS 754.68 on oil cakes. In the first step, the best substrate was chosen from four oil cakes, namely soybean, sunflower, olive, and colza oil cakes, of which sunflower oil cake showed the highest yield. In the next step, screening tests were performed using the Plackett‐Burman design. Seven variables (substrate particle size, moisture content, time, temperature, yeast extract, glucose, and glutamate) were investigated (each taking values of +1 and −1). Among these variables, time, temperature, and substrate particle size significantly affected ARA production (*p* < .05), so they were further investigated in the optimization step. The optimal fermentation time, temperature, and substrate particle size calculated by response surface methodology were 8.75 days, 18.5°C, and 1.3 mm–1.7 mm, respectively. Under these conditions, *M. alpina* was predicted to produce 4.19 mg of ARA/g dry weight of substrate (DWS). The actual yield, determined in evaluation tests, was 4.48 ± 0.16 mg ARA/g DWS, which shows the accuracy of the model. In the final step, the effect of the aeration rate on producing ARA was investigated in a packed‐bed solid‐state fermenter under the determined optimal conditions. In this stage, the highest ARA yield was 10.13 ± 0.26 mg/g DWS, approximately double that of the optimization step, and this confirms that aeration increases ARA production by *M. alpina*.

## Graphical abstract

The present study optimizes arachidonic acid production by the soil fungus *Mortierella alpina* CBS 754.68 on oil cakes. The research was performed in five stages: the selection of a suitable substrate, the screening and optimization of the most effective variables with two statistical methods, a response surface methodology assessment, and an investigation of aeration in a packed‐bed solid‐state fermenter.

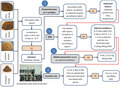

## 1 INTRODUCTION

Omega‐3 and omega‐6 fatty acids are essential to human health (Abedi & Sahari, 2014). The conversion efficiency of linoleic acid (LA) and α‐linolenic (ALA) acid to arachidonic acid (ARA), eicosapentaenoic acid (EPA), and docosahexaenoic acid is low in the human body, so it is recommended that we include them in our diet plan (Abedi & Sahari, 2014). ARA, an omega‐6 polyunsaturated fatty acid (PUFA) 20:4( ω‐6), is especially important for infants. The body uses it to produce regulatory molecules such as prostaglandins and thromboxanes (Certik & Shimizu, 1999; Dieter, 1994). ARA also has important roles in the central nervous system and in increasing the immune response (Mamani et al., 2019).

Most PUFAs, including ARA, are mainly obtained from fish oil and pig liver, but among the best sources of ARA are microorganisms. Because the sensorial acceptance of these animal oils is low on account of the taste, odor, and high cholesterol level (Hwang et al., 2005), microbial PUFAs, especially those produced by fungi, are more desirable for our diet (Cheng et al., 1999).

To date, PUFAs usually have been produced from submerged cultures of a range of fungi, including some species of *Mortierella* such as *M. alliacea* (Papanikolaou et al., 2004), *M*. *alpina* (Jang et al., 2000), and *M. isabellina* (Aki et al., 2001); *Mucor rouxii* (Jangbua et al., 2009); some *Cunninghamella* species such as *C. elegans* and *C. echinulata* (Certik et al., 2006); and *Saccharomyces cerevisiae* (Gema et al., 2002). Some bacteria can also produce PUFAs but are not appropriate for mass production because of the toxicity of the products and the slow growth rate of these bacteria (Ratledge, 2004). Among microorganisms, *M. alpina* seems to be the best option for producing some PUFAs such as ARA (Ferreira et al., 2020). Singh and Ward (1997) showed that *M. alpina* ATCC 32,222 can synthesize up to 11 g/L of ARA in 11 days. Other studies showed that this fungus can produce up to 20 g/L of triacylglycerol in culture broth (Kikukawa et al., 2018).

Many studies have investigated PUFA production by submerged *Mortierella* cultures, particularly the production of ARA, LA, and EPA (Aki et al., 2001; Zhu et al., 2003). Glycerol or glucose is usually used as a carbon source for PUFA production by microbial cultures (Zhu et al., 2003). Although submerged fermentation is easily implemented, submerged cultures demand high energy and produce more waste than solid‐state cultures (Jang & Yang, 2008).

To become economically competitive, PUFA production should be upscalable to the rural level. It can be achieved in solid‐state fermentation (SSF) because the cost of growing microorganisms will be reduced, whereas the product yield will be increased (Jang & Yang, 2008); also, it reduces costs because of the use of cheap substrates such as agricultural byproducts (Darabzadeh et al., 2019).

Some microorganisms can produce PUFAs on solid substrates. For example, the fatty acid γ‐linolenic acid (GLA) is produced by *Thamnidium elegans* growing on cereals (Certik et al., 2006). *Mucor rouxii*, *Cunninghamella elegans*, *Geotrichum candidum*, and some other microorganisms could also synthesize GLA in SSF (Conti et al., 2001; Fregolente et al., 2009; Jangbua et al., 2009). *Pythium ultimum* grown on solid substrates produced ARA and EPA (Stradansky et al., 2000).

Agricultural wastes are appropriate cultures for PUFA production. Experimenting with the growth of *M. alpina* on the bran of cereals, Jang et al. (2000) reported that the best substrate for this microorganism to produce ARA is rice bran. This fungus can also produce other microbial lipids, such as GLA, by growing on cereals (Cheng et al., 1999). Oil cakes are among the major cheap oil and fat factory byproducts that can be utilized as good media to produce ARA.

This paper investigates the substrate suitability of four oil cakes, namely soybean, sunflower, olive, and colza oil cakes, for the production of ARA by *M. alpina*. The variables that chiefly affect ARA synthesis on the chosen substrate are then assessed by the Plackett‐Burman (PB) screening design. Optimization is performed using response surface methodology (RSM), which has been shown to be a suitable method for this step (Malaiwong et al., 2016). Finally, the effect of the aeration rate on ARA production by this microorganism is investigated in a packed‐bed solid‐state fermenter under the optimal conditions established from the RSM step.

## 2 MATERIALS AND METHODS

### 2.1 Substrates

Native oil factories were the main sources for obtaining oil cakes. The initial ARA contents of soybean, sunflower, olive, and colza oil cakes were 0.02, 0.00, 0.00, and 0.014 mg/g of oil cake dry weight, respectively, and the corresponding moisture contents were 0.1, 0.1, 0.09, and 0.08 on a wet basis.

### 2.2 Media and culture conditions

*M. alpina* CBS 754.68 was purchased from Centraalbureau voor Schimmelcultures (CBS; the Netherlands). To obtain suitable cultures ready for sporulation, it was initially grown on slants and later on plates of malt agar at 22°C (Rocky et al., 2011). By growing the fungus on Czapek‐Dox agar plates at 28°C for 17 days, we obtained spores. After incubation, each plate was washed with 3 ml of a diluting solution (1 g of bacteriologic peptone dissolved in a saline solution, with the latter prepared from 8.5 g NaCl in 100 ml of distilled water) and filtered to remove the mycelia; this yielded a pure spore suspension. This pure spore suspension was blended with 23% v/v glycerol (to a volume of 1 ml) and retained in 1.5‐ml microtubes in a freezer maintained at −20°C. Every 1 ml of stored spore suspension contained $10^{5}$ spores (Rocky et al., 2011).

The first stage of SSF, choosing the best growth substrate, was performed in 500‐ml flasks. As a substrate, each flask contained 10 g of oil cake (sterilized for 30 min at 121°C). To ensure the absence of free water in the substrates, the moisture content was adjusted to a 50% wet basis for soybean, sunflower, and colza oil cakes and to 10% for olive oil cake. Each flask before being incubated at 20°C for 6 days was inoculated with 1 ml of the spore suspension, and this was followed by 12°C for another 6 days (Jang et al., 2000).

### 2.3 PB screening design

The PB screening design was applied to the selected oil cake in this step, with each variable scored as low (L) or high (H). The design contained 7 variables tested in 8 runs: substrate particle size (A), moisture content (B), time (C), temperature (D), yeast extract (E), glucose (F), and glutamate (G). Experiments were performed in 500‐ml flasks containing 10 g of substrate. All experiments were performed in triplicate, and the mean yields are reported as the results. The high and low levels of each variable are listed in Table 1. Results were statistically analyzed using Minitab 16 software (Minitab, Inc., State College, Pennsylvania), as it has been shown to be suitable software for analyzing data and to provide an effective way to input statistical data and receive patterns (Rocky et al., 2011).

**TABLE 1:** Levels of the 7 experimental variables included in the Plackett‐Burman design

Variables	Unit	Experimental values	
		Lower level	Higher level
A	mm	0.2–0.6	1–1.4
B	%	50	70
C	Day	2	6
D	°C	12	20
E	g/l	0	10
F	g/l	0	20
G	g/l	0	1

Abbreviations: A, substrate particle size; B, moisture content; C, time; D, temperature; E, yeast extract; F, glucose; G, glutamate.

### 2.4 Response surface methodology

A central composite design (CCD) via RSM was implemented to optimize ARA production in the culture medium. Each 500‐ml flask containing 10 g of the wet substrate was inoculated with $10^{5}$ spores after sterilization at 121°C for 30 min. The inoculated flasks were incubated at various temperatures and for various times (listed in Table 2). This step identified three important factors of ARA production: time, incubation temperature, and substrate particle size (see Table 2). A CCD including 18 runs, of which four were set at the central point, was used. All variables were adjusted to the central coded value (considered to be zero). Results were analyzed using the following second‐order polynomial equation:

$$Y=\alpha_{0}+\sum_{j=1}^{k}\alpha_{j}X_{j}+\sum_{j=1}^{k}\alpha_{\mathrm{jj}}X_{j}^{2}+\sum \sum_{i<j}\alpha_{\mathrm{ij}}X_{i}X_{j}$$

where Yis the predicted response, $X_{i}$and $X_{j}$are independent variables, $\alpha_{0}$is the offset term, $\alpha_{j}$is the j ^th^ linear coefficient, $\alpha_{\mathrm{jj}}$is the quadratic coefficient, and $\alpha_{\mathrm{ij}}$is the interaction coefficient.

**TABLE 2:** Levels of the experimental variables utilized in the RSM approach

Variables	Units	Symbol	Variable level		
			−1	0	+1
Particle size	mm	X_1_	0.6–1	1.2–1.6	1.8–2.2
Time	Day	X_2_	3	6	9
Temperature	°C	X_3_	16	20	24

Abbreviation: RSM, response surface methodology.

To draw the contour and three‐dimensional graphs, the statistical software package Design Expert 9.0.0 (Stat‐Ease, Inc., Minneapolis, Minnesota) was used for the regression analysis of the experimental data. This software is a strong tool for performing a CCD for a combination of factors and delivers contour plots, and this helps in understanding the effect of optimal points (Rocky et al., 2011).

### 2.5 Model assessment

To assess the optimal variables predicted by RSM, 10 g of the wet substrate (at the optimal substrate particle size) was added to 500‐ml flasks and sterilized for 30 min at 121°C. Each flask was inoculated with $10^{5}$ spores after cooling and then incubated at the optimal time and temperature.

### 2.6 Aeration rate effect on producing ARA in a packed‐bed solid‐state fermenter

**FIGURE 1:**
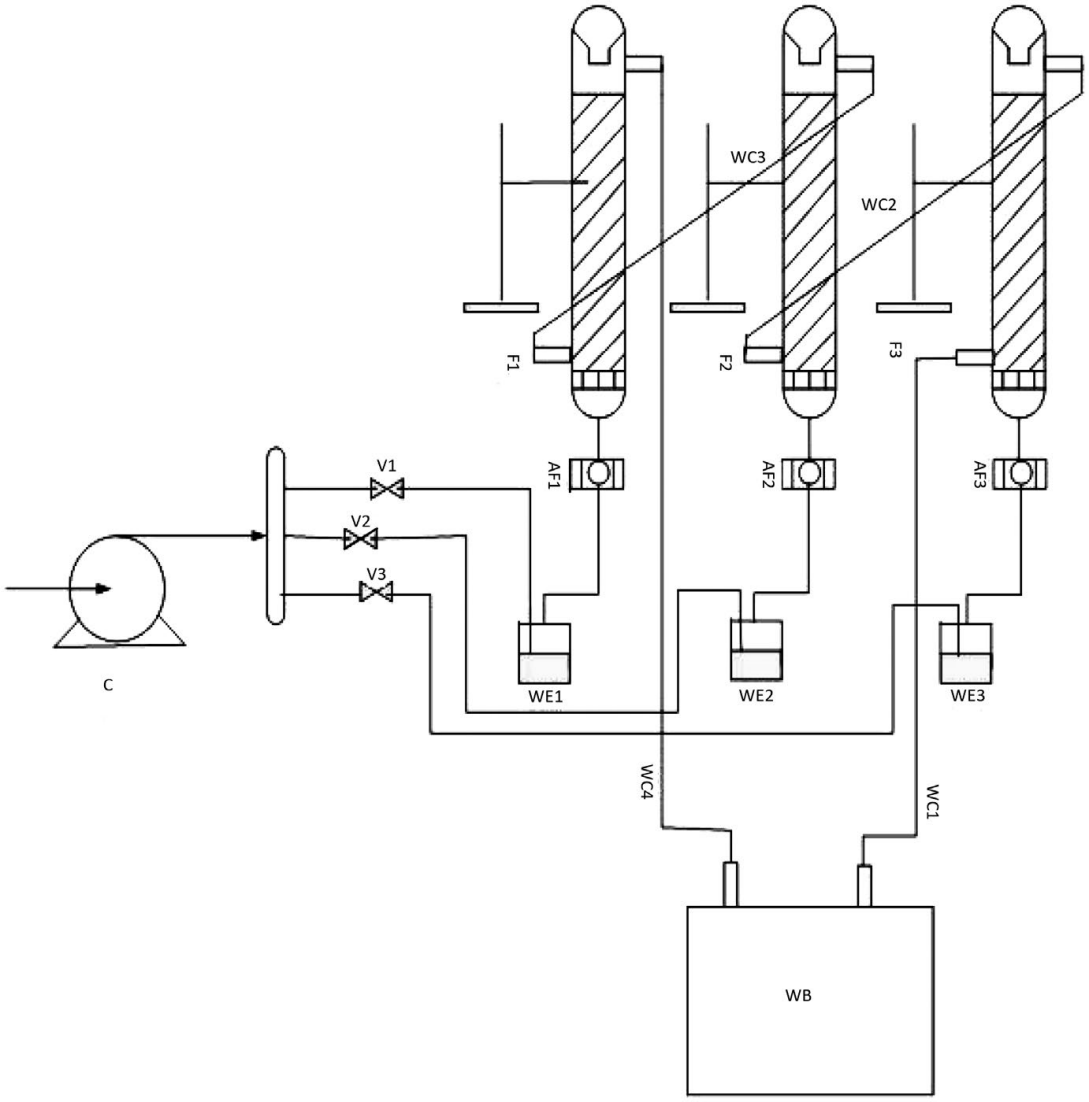
Schematic diagram showing the components of the packed‐bed fermenter: (C) aquarium compressor, (V_1_, V_2_, V_3_) entering air valves, (WE_1_, WE_2_, WE_3_) air‐wetting dishes (flasks), (AF_1_, AF_2_, AF_3_) air filters, (F_1_, F_2_, F_3_) fermenters, (WC_1_, WC_2_, WC_3_) water circulating vessels, and (WB) water bath

A packed‐bed solid‐state fermenter was prepared under the optimal conditions determined from RSM (Figure 1). The substrate was sterilized for 30 min at 121°C, inoculated with $10^{5}$ spores, and incubated for a 6‐day period at 12°C to allow primary growth. Next, a mixture of 5 g fermented substrate and 35 g of sterilized noninoculated substrate was added to the packed‐bed solid‐state fermenter. Fermentation was performed at the optimal time and temperature determined from RSM.

The aeration rates were 0.110, 0.320, and 0.530 L/ min. The air was passed over flasks filled with distilled water and gravel to become saturated before entering the fermenter. The fermenter temperature was kept constant at the optimal point obtained in the previous step by a circulator bath. A schematic picture of the fermenter is illustrated in Figure 1.

### 2.7 Lipid extraction and determination of the ARA content by gas chromatography

In each step of the experiment, the ARA content of the fermented oil cakes was determined by the extraction of lipids from the fermented oil cakes, then fatty acid derivation, and finally the injection of the derived samples into a gas chromatography (GC) machine (Metcalf et al., 1966). To extract lipids, the oil cakes were dried in an oven at 80°C for 24 hr, and then, 3 ml of N‐hexane as a solvent was added to 1 g of the dried substrate in a test tube (Folsch et al., 1957). To optimize the extraction, test tubes were put into an ultrasound bath (Jang et al., 2000). The solvent that contained extracted lipids was poured into a clean test tube and evaporated by injecting nitrogen, which is a neutral gas; hence, only lipids remained in the test tube (Rocky et al., 2011). As fatty acids are not volatile, they should be derived to their methyl ester forms, which are volatile and can be injected into the GC machine. Thus, 5 ml of 2% methanolic sodium hydroxide and then 1 ml of pentadecanoic acid (as an internal standard) were added to each test tube, and the tube was put into boiling water for 10 min. After cooling, 2.175 ml of boron trifluoride (BF_3_) was added to each tube, and the tube was put into boiling water again for 3 min. After cooling, 1 ml of N‐hexane solvent and finally 1 ml of saturated sodium chloride were added to each tube, and they were stirred. After a biphasic solution was obtained, the upper phase, which contained methylated fatty acids and solvent, was poured into a 1.5‐ml vial as the final sample to be injected into the GC machine (Metcalf et al., 1966).

A GC machine (model: Unicam 4600, England) was used to determine the fatty acid composition of the oil cakes. Each sample was injected into the machine, while nitrogen was used as a carrier gas. The fatty acid composition of the samples was determined on the basis of the retention time of the fatty acid compared with the retention time of the internal standard. The machine used a capillary column with a length of 30 m and a flame ionization detector. For each test, 0.2 µl of the sample was injected into the machine. The GC program set 160°C as the initial temperature of the column for 6 min; the temperature was increased to 180°C for 9 min and then increased to 190°C for 25 min (at a speed of 20°C/min). The injector temperature was 250°C, and the detector temperature was 300°C. The fatty acid amount (mg/g of dry weight of substrate [DWS]) was calculated as follows:

$$E=\frac{A\times \left(\frac{B}{C}\right)}{D}$$

where *A* is the area of the unknown peak, *B* is the concentration of the internal standard, *C* is the area of the internal standard peak, *D* is the sample weight (g), and *E* is the fatty acid amount (Rocky et al., 2011)

## 3 RESULTS AND DISCUSSION

### 3.1 Substrate selection

In the primary step, sunflower oil cake (with a yield of 1.14 mg of ARA/g DWS) was identified as the most proper substrate for producing ARA, and it was followed by oil cakes of soybeans (0.53 mg of ARA/g DWS), colza (0.38 mg of ARA/g DWS), and olives (0.066 mg of ARA/g DWS). The high yield of sunflower oil cakes may be attributed to the fatty acid composition of this substrate, where up to 90% of the sunflower oil is composed of unsaturated fatty acids (linoleic and oleic acids) (Malek, 2000). Media with a high PUFA content, especially LA, are known to stimulate ARA production by *M. alpina* (Jang, 2000). The LA content of sunflower oil is 55%–80% of the total fat, and this is followed by soybean oil (45%–55%), colza oil (15%–22%), and olive oil (8%–10%) (Certik & Adamechova, 2009). This shows that their oil cakes have the same fatty acid composition, which stimulates ARA production as mentioned previously. Using some oil cakes to produce proteins and PUFAs by *M. alpina* was previously studied by Ferreira et al.(2020), and their work showed that oil cakes are suitable media for this fungus to synthesize these nutrients.

### 3.2 PB screening design

According to the aforementioned description, seven variables were selected: substrate particle size, moisture content, time, temperature, yeast extract, glucose, and glutamate. This step evaluated the importance of these variables to ARA production by the PB design via a Student *t* test. Responses with a *P* value below 0.05 were regarded as statistically significant. Table 3 shows the PB experimental design and the results for the different variables.

**TABLE 3:** Experimental Plackett‐Burman design and obtained results (mg ARA/g DWS)

Experiment	A	B	C	D	E	F	G	Yield
1	H	L	H	L	L	H	H	3.93
2	H	H	L	L	H	L	H	2.81
3	H	H	L	H	L	H	L	3.11
4	L	H	H	H	L	L	H	3.36
5	H	L	H	H	H	L	L	4.18
6	L	H	H	L	H	H	L	2.65
7	L	L	L	H	H	H	H	2.43
8	L	L	L	L	L	L	L	1.64

Abbreviations: A, substrate particle size; ARA, arachidonic acid; B, moisture content; C, time; D, temperature; DWS, dry weight of substrate; E, yeast extract; F, glucose; G, glutamate; H, higher level; L, lower level.

A positive/negative effect of the tested variable indicates that ARA production is greater/smaller at the high levels of the tested variable versus the low levels. Among these variables, time, substrate particle size, and temperature demonstrated significant positive effects (Table 4).

**TABLE 4:** Estimated effects of the 7 variables on APA production and corresponding regression coefficients in the Plackett‐Burman design

Variables	Effect	Regression coefficient
A	0.98	0.98
B	−0.06	−0.03
C	1.03	0.51
D	0.51	0.25
E	0.007	0.003
F	0.03	0.01
G	0.23	0.11

Abbreviations: A, substrate particle size; B, moisture content; C, time; D, temperature; E, yeast extract; F, glucose; G, glutamate.

A larger substrate particle size (1 mm–1.4 mm) favored ARA production, probably because the hard, stiff cakes formed by smaller particle sizes inhibited suitable aeration and fungal growth. According to Pandey et al. (2000), a decreased interparticle pore size prevents oxygen molecules from reaching the mycelia of the fungi, and this inhibits fungal respiration. ARA production by *M. alpina* was enhanced after a 12‐day incubation at 20°C. Some of the enzymatic paths by which LA is converted into ARA in the mycelium are very slow; thus, a prolonged incubation time can have a positive effect (Gema et al., 2002; Stradansky et al., 2000). Increasing the temperature also enhanced the ARA yield. At 12°C, ARA production was reduced, whereas EPA production was enhanced. This suggests that an intracellular enzyme activated at low temperatures catalyzes the conversion of ARA to EPA (Cheng et al., 1999). Other variables showed no effect on the production of ARA.

### 3.3 Optimization of effective variables for ARA production using RSM

This step aimed to establish a mathematical model for predicting the outcome of the process. To predict the optimal points based on the analyzed areas, RSM can analyze interactive effects of the variables (Wu et al., 2017). The variables that significantly affected ARA production in the preceding step, namely time, temperature, and substrate particles size, were selected for investigation in this procedure.

Small substrate particles exerted a negative effect on ARA production: BP analysis confirmed a positive association between larger particle size and ARA production. Thus, a larger minimum particle size was selected in this step. Because of the significant positive effect of time on ARA production, the central time point was set as the maximum fermentation time identified by PB (6 days). Because the temperature also exerts a significant positive effect on ARA production, the minimum and central temperatures were set to 16°C and 20°C, respectively. The variable levels (+1, −1, or 0) selected in the optimization step are shown in Table 5.

**TABLE 5:** Experimental design and ARA yields obtained with the central composite design

Experiment	Substrate particle size	Temperature	Time	ARA concentration (mg/g DWS)
1	−1	−1	−1	2.43 ± 0.09^a^
2	−1	−1	1	2.99 ± 0.23
3	−1	1	−1	2.08 ± 0.14
4	−1	1	1	2.67 ± 0.11
5	1	−1	−1	2.21 ± 0.15
6	1	−1	1	4.04 ± 0.16
7	1	1	−1	1.98 ± 0.12
8	1	1	1	2.62 ± 0.17
9	0	0	−1	2.76 ± 0.08
10	0	0	1	4.28 ± 0.21
11	0	−1	0	3.24 ± 0.11
12	0	1	0	3.01 ± 0.10
13	−1	0	0	3.32 ± 0.11
14	1	0	0	2.83 ± 0.06
15	0	0	0	3.64 ± 0.13
16	0	0	0	3.54 ± 0.11
17	0	0	0	3.76 ± 0.12
18	0	0	0	3.51 ± 0.14

Abbreviations: ARA, arachidonic acid; DWS, dry weight of substrate.

^a^ Mean ± standard deviation (*SD*).

On the basis of the outcome of the preceding step, the moisture content was set at 50%, and glutamate, which exerted a slightly positive effect on ARA production, was added at 1 g/L. Glucose and yeast extract, which scarcely affected ARA production, were not added to the culture medium.

In the optimization tests, ARA production was maximized at 4.28 ± 0.21 mg/g DWS in experiment number 10 under the following conditions: an incubation time of 9 days (after 6 days of incubation at 20°C), an incubation temperature of 20°C, and a particle size of 1.2 mm–1.6 mm.

The lowest ARA yield was 1.98 ± 0.12 mg/g DWS in experiment number 7 under the following conditions: an incubation time of 3 days (after 6 days of cultivation at 20°C), an incubation temperature of 24°C, and a particle size of 1.8 mm–2.2 mm. The optimization experimental results are summarized in Table 5.

The results of an analysis of variance of the obtained data are presented in Table 6. The coefficient of determination (*R* ^2^) was 0.92, and the adjusted *R* ^2^ value was 0.83. The values indicate a strong agreement between the experimental data and the regression model prediction. The *F*‐test probability and insignificant lack of fit show that the model accurately describes the experimental data and can therefore be used to optimize the process.

**TABLE 6:** Variance analysis in the ARA regression model derived from RSM

Factor	SS	DF	MS	*F* value	*p* value
Model	6.94	9	0.77	10.19	.0017
Time	2.64	1	2.64	34.9	.0004
Temperature	0.65	1	0.65	8.59	.019
Particle size	3.61	1	3.61	0.048	.8326
Temperature × time	0.17	1	0.17	2.22	.1744
Particle size × time	0.22	1	0.22	2.88	.1283
Particle size × temperatures	0.12	1	0.12	1.59	.2434
Time × time	4.785	1	4.785	0.063	.8078
Temperature × temperature	0.52	1	0.52	6.84	.0309
Particle size × particle size	0.64	1	0.64	8.49	.0195
Remainder	0.61	8	0.076		3.24
Lack of fit	0.57	5	0.11	8.89	.0509
Pure error	0.038	3	0.013		
Total correlation	7.55	17			

Note

*R* ^2^ = 0.92; adj *R* ^2^ = 0.83.

Abbreviations: ARA, arachidonic acid; DF, degrees of freedom; MS, mean squarek RSM, response surface methodology; SS, sum of squares.

The regression model used for predicting ARA production by *M. alpina* is given as follows:

(1) $$Y=-11.26052+0.35903X_{1}+1.16256X_{2}+3.73933X_{3}+0.012083X_{1}X_{2}+0.091667X_{1}X_{3}-0.051042X_{2}X_{3}-4.66931X_{1}^{2}-0.027314X_{2}^{2}-1.35284X_{3}^{2}$$

where Y denotes the predicted ARA yield and $X_{1}$, $X_{2}$, and $X_{3}$ denote time, temperature, and substrate particle size, respectively.

As shown in Table 6, time and temperature showed a significant effect on the ARA yield in this step, whereas the effect of the substrate particle size was negligible (*p* > .05). Significant second‐order effects were observed only for temperature and substrate particle size.

**FIGURE 2:**
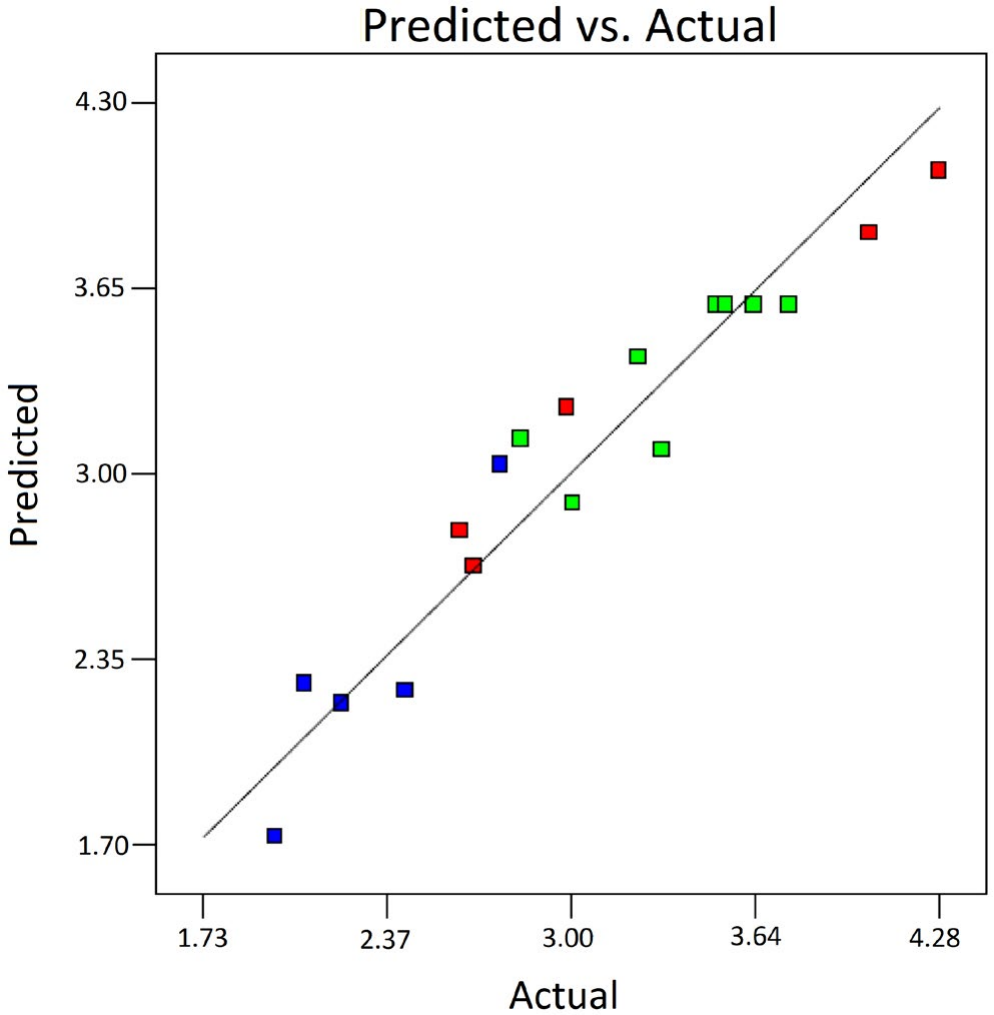
Yield correlation: data predicted by the model versus experimental data

The data predicted by the model are compared with the experimental data in Figure 2. As the actual data are concentrated around the predicted data line, it is demonstrated that the model was able to predict the experimental data with high accuracy.

To determine the variable levels that maximize ARA production, we made contour and three‐dimensional plots by plotting the response (ARA yield) against each of the two independent variables and fixing the third variable constant at its central (zero) level.

**FIGURE 3:**
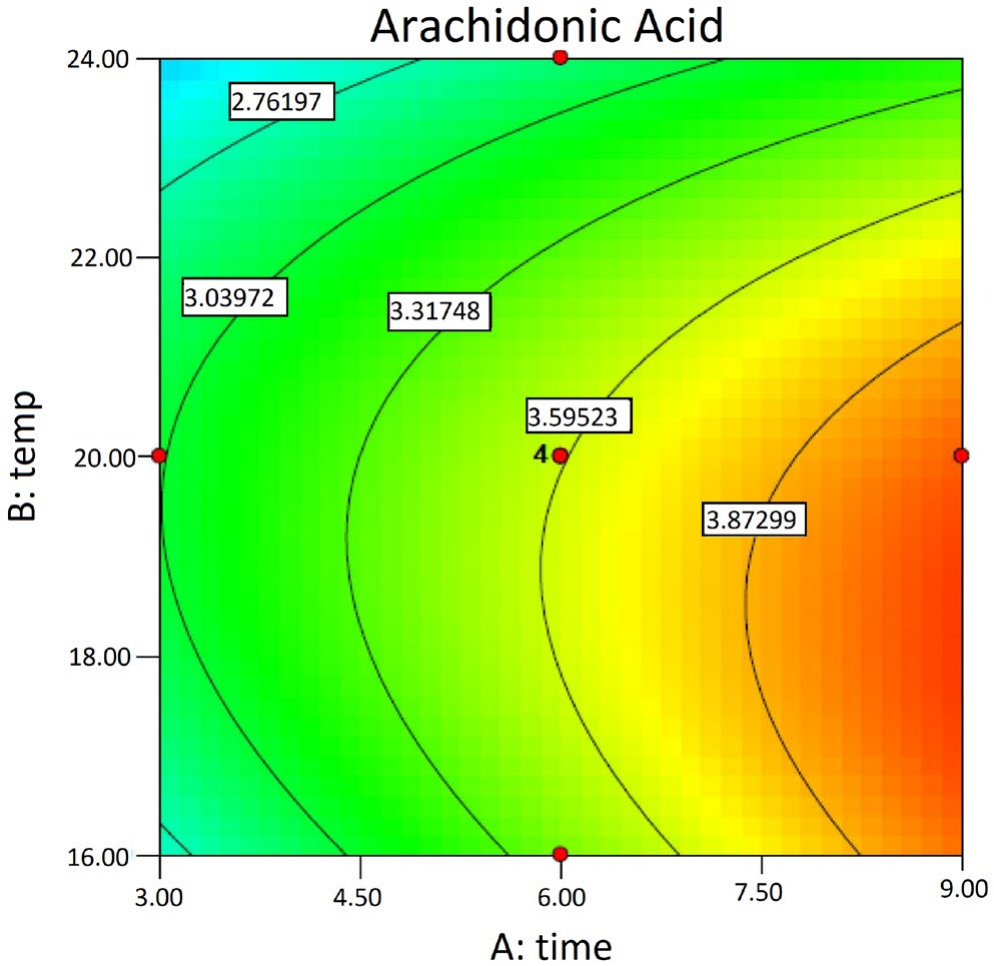
Contour plot of temperature and time effects on the ARA yield (mg/g dry weight of substrate). The substrate particle size is fixed at its central level. ARA, arachidonic acid; DWS, dry weight of substrate

Figure 3 illustrates a contour plot of time and temperature effects on the ARA yield at a fixed (central‐level) substrate particle size. Increasing the incubation temperature and time (after the primary 6 days) within the ranges of 16°C–20°C and 7.5–9 days, respectively, invoked an increase in ARA production by *M. alpina*. Furthermore, a significant second‐order effect of temperature was observed. ARA levels are low in short‐time fermentation and increase with increasing fermentation time.

**FIGURE 4:**
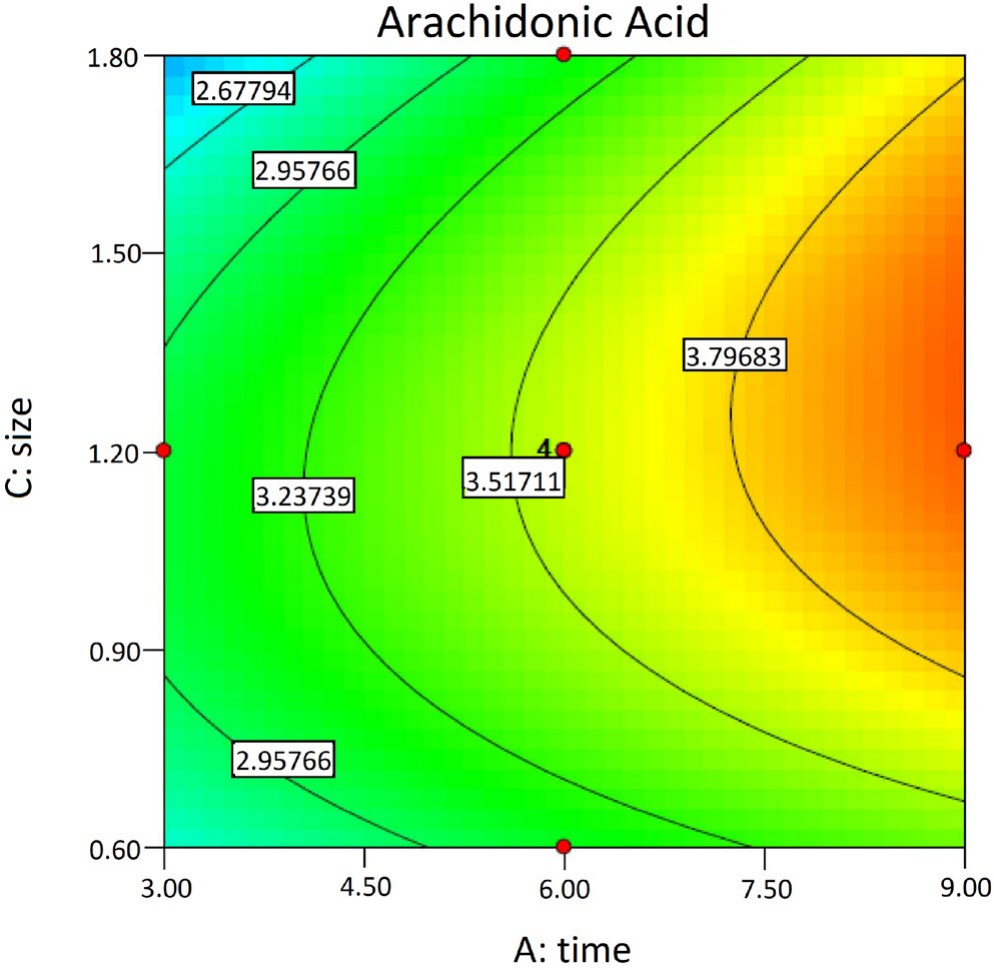
Contour plot of time and substrate particle size effects on the ARA yield (mg/g DWS). The temperature is fixed at its central level. ARA, arachidonic acid; DWS, dry weight of substrate

Figure 4 shows a contour plot of time and substrate particle size effects on the ARA yield, with the temperature fixed at its central level. In this case, limiting the substrate particle size to 1–1.5 mm and increasing the time within the range of 7.5–9 days (after the primary 6 days) increased ARA production by *M. alpina*. Thus, although ARA production was enhanced by increasing the fermentation time at a fixed temperature, increasing or decreasing the substrate particle size produced no effect, and ARA production was maximized at the average size of the substrate particles. According to Figure 4, the square of the substrate particle size exerts a significant effect on ARA production, and this is consistent with the data presented in Table 6.

**FIGURE 5:**
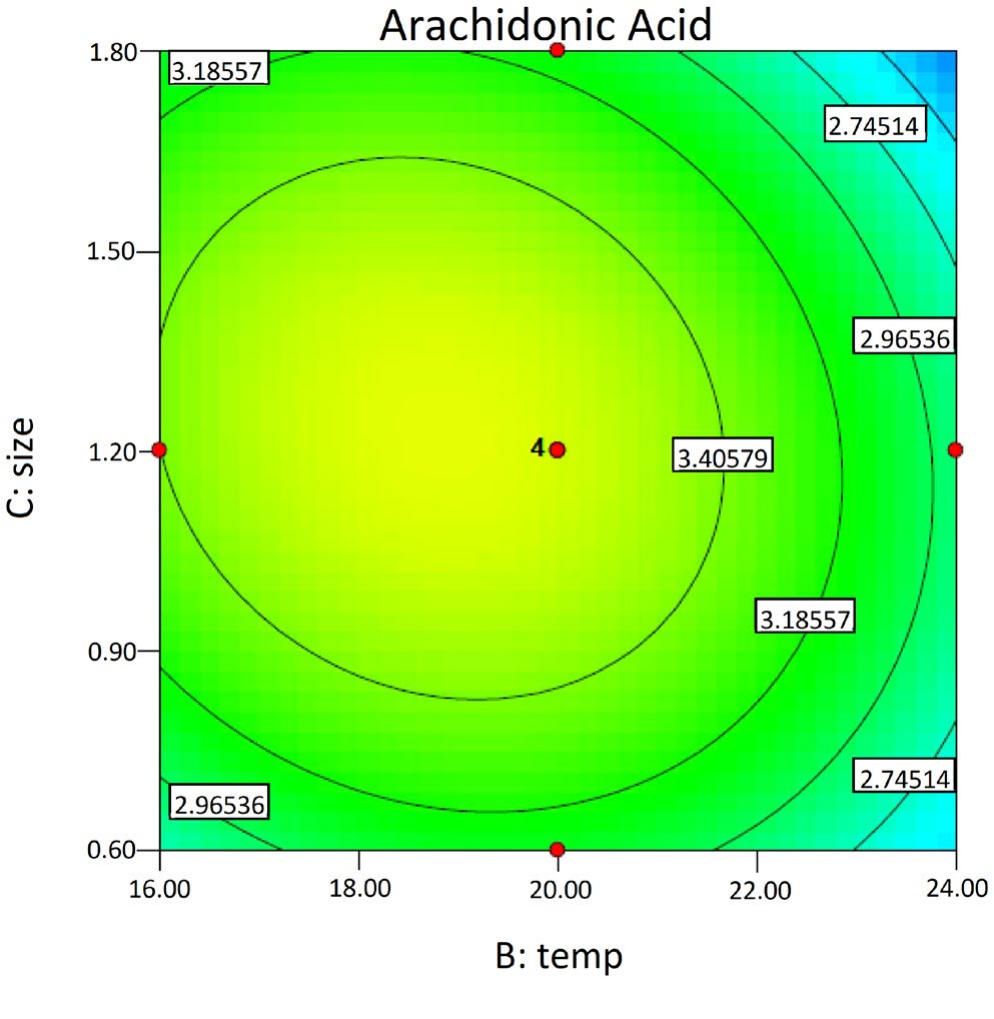
Contour plot of temperature and substrate particle size effects on the ARA yield (mg/g DWS), with time fixed at its central level. ARA, arachidonic acid; DWS, dry weight of substrate

Figure 5 presents a contour plot of temperature and substrate particle size effects on the ARA yield (mg/g DWS), with time fixed at its central level (6 days after the primary 6 days). As it shows, the temperature has an important effect on ARA production, which is positive up to about 20°C and is negative at higher temperatures. The substrate particle size does not show an important effect on ARA production, and this is consistent with the data presented in Table 6

In this step, the optimal conditions for producing ARA were numerically evaluated with Design Expert 9 software. Design Expert 9 calculated the optimum fermentation time, fermentation temperature, and substrate particle size as 8.75 days, 18.5°C, and 1.3 mm–1.7 mm, respectively. Under these conditions, the predicted ARA production yield by *M. alpina* is 4.19 mg of ARA/g DWS.

### 3.4 Model assessment

To evaluate the model acquired by RSM, an experiment was conducted in triplicate under the identified optimal conditions. In this experiment, the mean yield obtained by *M. alpina* was 4.483 ± 0.16 mg ARA/g DWS, which was satisfactorily close to the yield predicted by the model. Thus, the model acquired by RSM was able to predict the incubation behavior and optimize the production yield of *M. alpina*.

### 3.5 Evaluation of the aeration effect on ARA synthesis in a packed‐bed solid‐state fermenter

To evaluate the influence of aeration on ARA synthesis by *M. alpina*, the organisms were cultivated in a packed‐bed solid‐state fermenter under the optimized conditions determined by the RSM model, while the aeration rate was varied. The mean yields (all experiments were performed in triplicate) were 10.13 ± 0.26, 4.93 ± 0.19, and 2.18 ± 0.22 mg ARA/g DWS at aeration rates of 0.11, 0.32, and 0.53 L/min, respectively.

The yield obtained at the optimal aeration rate of 0.110 L/min is 2 times higher than that obtained under the optimal conditions without aeration. Aeration increases microbial growth by supplying essential oxygen. It also encourages PUFA production because the unsaturation of fats in cells similarly requires molecular oxygen (Aiba et al., 1973; Lambraki et al., 1994). It can increase the ARA yield in the fed‐batch fermentation process up to 25% (Wu et al., 2017).

The yield was reduced at higher aeration, probably because vigorous aeration damaged the microorganism's mycelia. On the contrary, because *M. alpina* is a slow‐growing organism, the wet air likely increased the substrate particle size and thereby decreased the size of the interparticle pores and prevented effective mass transfer.

## 4 CONCLUSION

From the results of this study of four oil cake substrates (sunflower, soybean, colza, and olive), the sunflower oil cake emerges as the best substrate to produce ARA by *M*. *alpina*, which yielded 1.14 mg of ARA/g DWS. The PB design showed that among other factors, the fermentation time, fermentation temperature, and substrate particle size have important positive effects on ARA production by *M. alpina*. The RSM design investigated these three factors to obtain optimal conditions, and a numerical analysis showed that the optimum fermentation time, fermentation temperature, and substrate particle size are 8.75 days, 18.5°C, and 1.3 mm–1.7 mm, respectively. The PB and RSM designs proved to be effective techniques for screening and optimizing the fermentation process. A model assessment showed that under the identified optimal conditions predicted by RSM, the mean yield obtained by *M. alpina* was 4.483 ± 0.16 mg ARA/g DWS, which was close to the amount predicted by RSM (4.19 mg of ARA/g DWS). Aeration at the rate of 0.11 L/min exerted a strong effect on ARA production by *M. alpina* and increased the yield up to 10.13 ± 0.26 mg ARA/g DWS, which is more than two times that obtained under the identified optimal conditions but without aeration.

## CONFLICT OF INTEREST

The authors declare no conflicts of interest.

## ETHICS APPROVAL

This article does not contain any studies with participants or animals requiring ethics approval.

## Data Availability

Data are available upon request from the authors.
